# On the Action and Uses of the Saliva

**Published:** 1839-01

**Authors:** 


ART. XII.—*De Salivâ ejusque vi et utilitate. Diss. Inaug.* Auct. G. VAN SETTEN.—*Groningæ*, 1837. 8vo. pp. 61.

*On the Action and Uses of the Saliva.* By G. VAN SETTEN, M.D.—*Groningen*, 1837. 8vo. pp. 64.

THIS production contains interesting results on those two points of the chemical history of the saliva that have of late been most warmly debated,—namely, its containing, or not, sulphocyanic acid, and the character of its reaction. The analysis was performed on the saliva of a healthy adult affected with salivary fistula, and, by the following phenomena, gave unquestionable evidence of the presence of the acid. The aqueous solution of the alcoholic extract of the saliva having been evaporated, and distilled with phosphoric acid, formed a fluid that, on the addition of perchloride of iron, exhibited the peculiar red tinge characteristic of sulphocyanuret of iron: when disappearing, the colour was restored by the addition of hydrochloric acid. Again, on adding to the distilled liquid a mixture of hydrochloric acid, chloride of potassium and barium, sulphate of baryta was formed; its acid resulting from the oxidation of the sulphur contained in the sulphocyanic acid. The result is rendered still more certain by the fact that the substance thrown down from the distilled fluid by the sulphates of iron and copper acquired, when washed and mixed with potassa, the same red tint on the addition of perchloride of iron.

We subjoin the analysis given by the author, which the reader will find to agree pretty closely with that of Gmelin. 100 parts consisted of

| | | |
|---|---|---|
| A. | Solid matter . . . . . . . . . . . . | 1·62 |
| B. | *a.* Matter precipitable by water from the alcoholic extract, probably phosphorous fat . . . . . . . . . .<br>*b.* Matter soluble in cold alcohol and in water, containing osmazome, sulphocyanic, chloride and acetate of potassa . . . . | 36·84 |
| | *c.* Matter precipitated by cooling from hot alcoholic solution, containing animal matter and an alkaline sulphate and chloride . | 4·72 |
| | *d.* Substance soluble in water alone, consisting of the salivary principle and some salts . . . . . . . . . . | 15·62 |
| | *e.* Substance insoluble in water and alcohol . . . . | 43·44 |

With respect to the reaction of the saliva, it was found

| | | |
|---|---|---|
| *acid,* | before breakfast . . . . | 17 times, |
| | after breakfast and dinner . . . | 25 .. |
| *alkaline,* | before breakfast . . . | 24 .. |
| | after breakfast and dinner . . | 15 .. |
| *neutral,* | before breakfast . . . | 9 .. |
| | after breakfast . . . . . | 10 .. |

From another series of experiments, Dr. Van Setten concludes that, in persons whose saliva is acid before breakfast, that fluid becomes alkaline during the process of eating, and after breakfast reassumes its acid reaction. In conjunction with Professor Sebastian, he made a number of trials which demonstrated, in confirmation of the assertions of Leuchs, Schwann, and others, the peculiar chemical action of the saliva on fecula.

